# Supplementary material for: Genome-Wide Characterization and Gene Expression Analyses of Malate Dehydrogenase (MDH) Genes in Low-Phosphorus Stress Tolerance of Chinese Fir (Cunninghamia lanceolata)
Source: Int J Mol Sci. 2023 Feb 23;24(5):4414. doi: 10.3390/ijms24054414 (PMC10003207; doi:10.3390/ijms24054414)
Supplement: Supplementary file 1 [file ijms-24-04414-s001.zip › TABLE S3.pdf]

TABLE S3|Putative motifs identified from ClMDH proteins using MEME.

The sequence logos were generated using WebLogo.

Motif name	Sequence logo	Frequence	Width	e-value
**Motif 1**	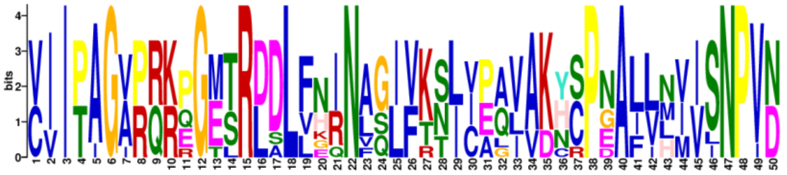 VIIPAGVPRKPGETRDDLFNINAGJVKSJVPAVAKHSPNALLNVISNPVB	0.750	50	4.10E-158
**Motif 2**	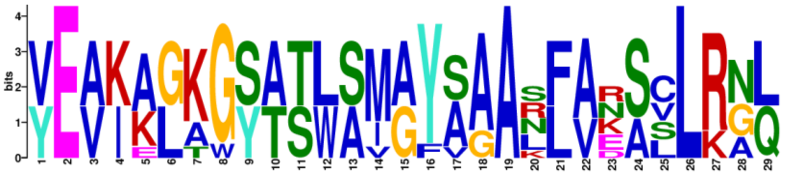 VEAKAGKGSATLSMAYAAARFAESCLRNL	0.750	29	6.80E-71
**Motif 3**	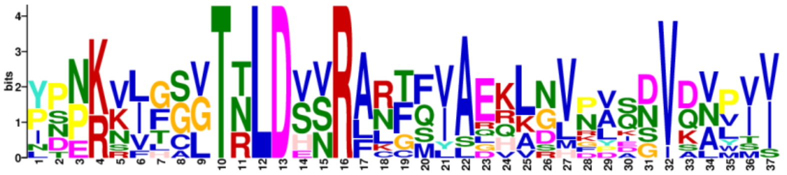 YPNKVJGSVTTLDSSRARTFIAEKLNVNVSDVDVPII	**0.917**	37	7.30E-73
**Motif 4**	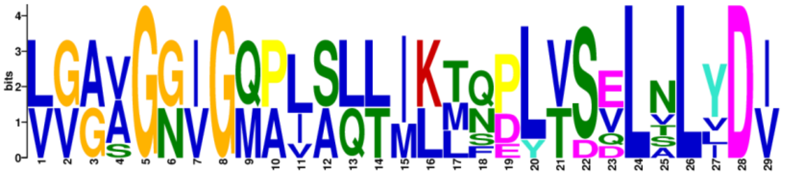 LGAAGGIGQPLSLLIKTQPLVSELNLYDI	0.583	29	2.00E-36

**Motif 5**	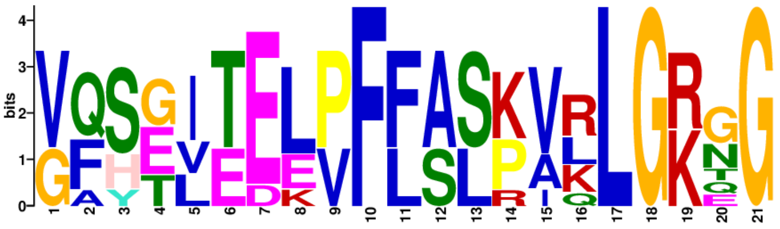<br>VQSEITELPFFASKVRLGKGG	0.667	21	2.80E-33
**Motif 6**	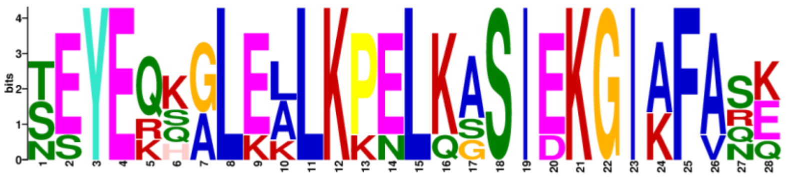<br>SEYEQKGLEALKPELKASIEKGIAFASE	0.417	28	2.60E-29
**Motif 7**	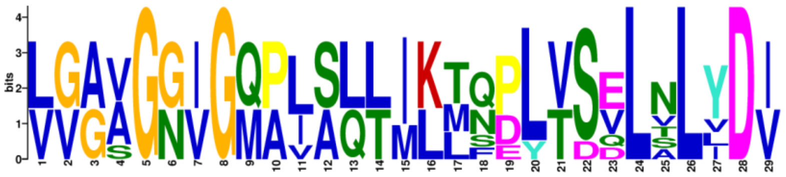<br>LRGVAADLSHAATPLPRTVIMGSDELEVS	0.833	29	8.80E-27
**Motif 8**	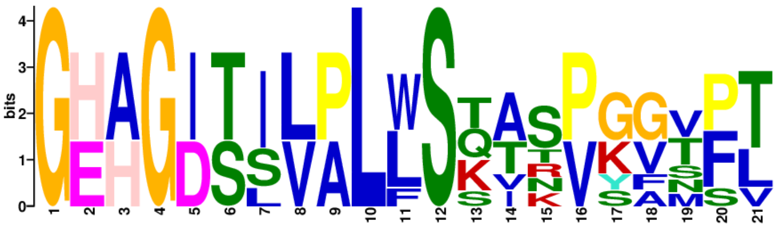<br>GHAGITILPLWSKASPGGTPT	0.583	21	6.40E-19
**Motif 9**		0.333	20	9.00E-13

	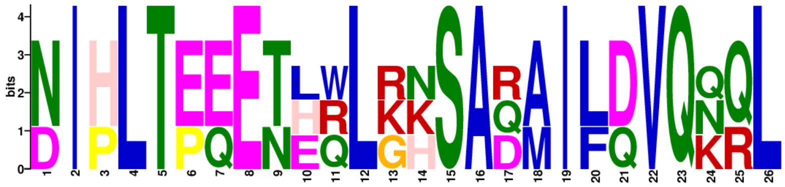<br>QIPYEPHPLEAJHQGVINSA			
**Motif 10**	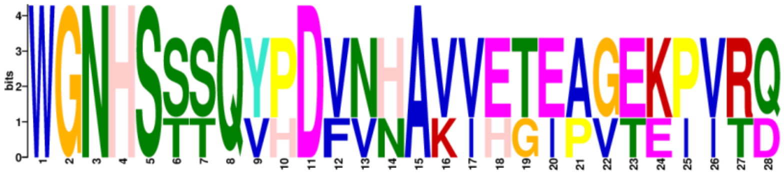<br>WGNHSSSQYPDVNHAVVETEAGEKPVRQ	0.250	28	2.70E-10
**Motif 11**	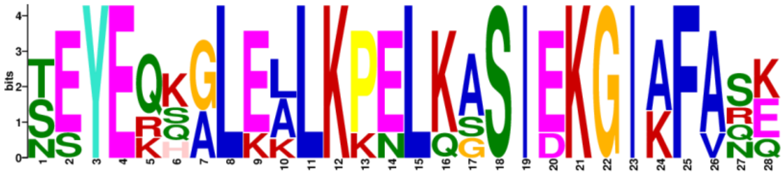<br>STVPIAAEVLKKAGV	0.417	15	5.20E-10
**Motif 12**	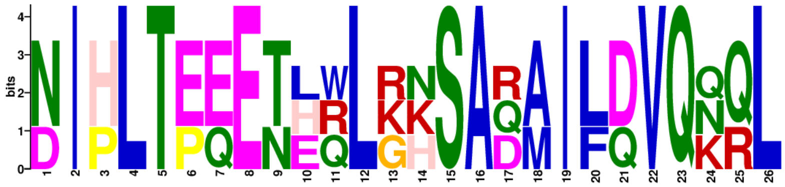<br>RRIHPVSVLAK	0.333	11	1.30E-08
**Motif 13**	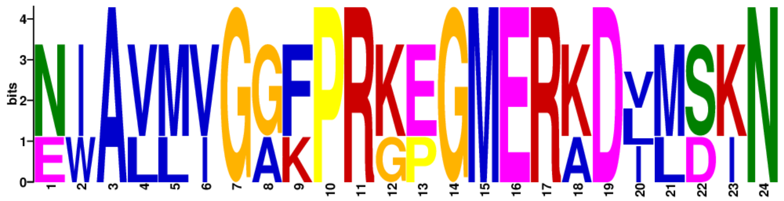<br>NIAVMVGGFPRKEGMERKDIMSKN	0.250	24	1.70E-07

**Motif 14**	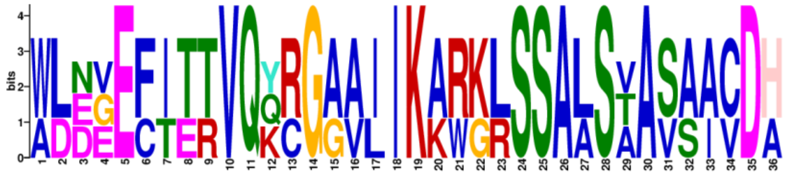 WLDGEFITTVQKRGAAIIKARKLSSALSVASAACDH	0.250	36	2.50E-07
**Motif 15**	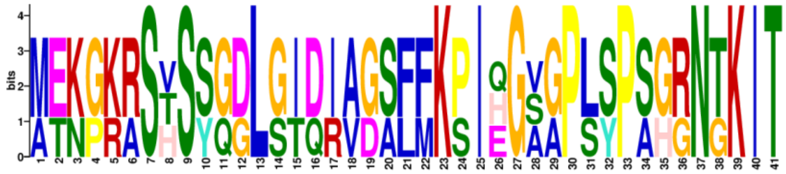 MEKGKRSVSSGDLGIDIAGSFFKPIQGAGPLSPSGRNTKIT	0.250	41	3.40E-07
**Motif 16**	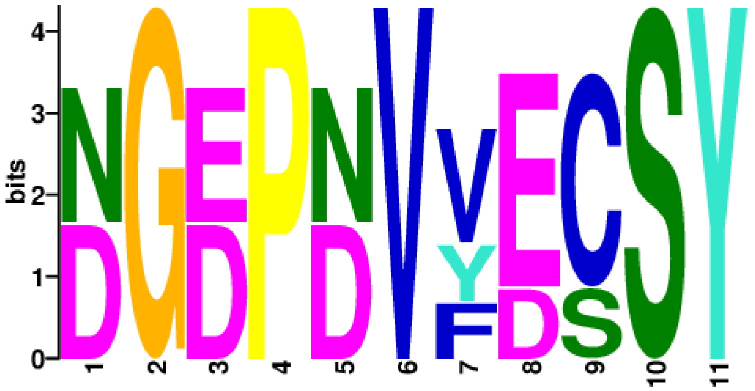 DGDPDVVECSY	0.333	11	6.00E-06
**Motif 17**	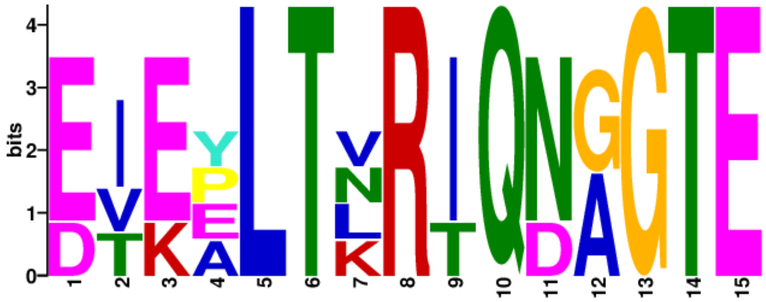	0.333	15	2.30E-05

	EIEPLTVRIQNGGTE			
**Motif 18**	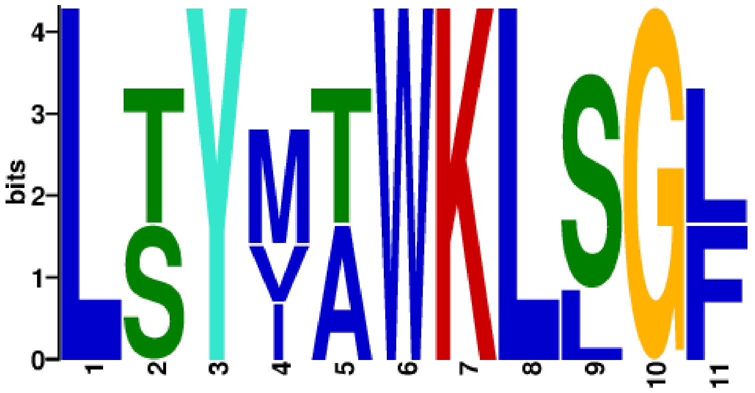 LSYMAWKLSGF	0.333	11	2.90E-05
**Motif 19**	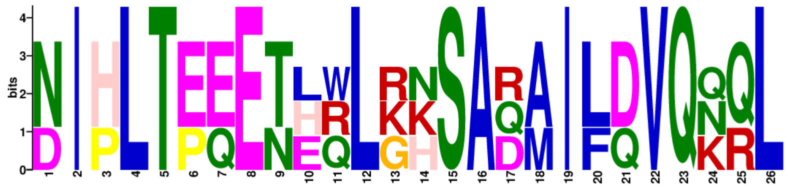 NIHLTEEETHRLKNSARAILDVQKQL	0.250	26	7.50E-05
**Motif 20**	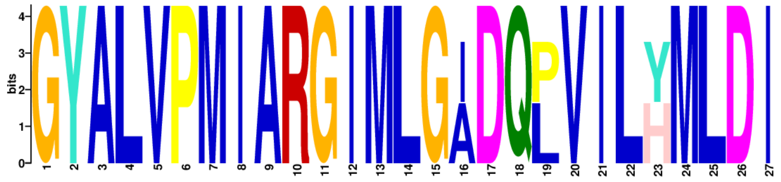 GYALVPMIARGIMLGIDQPVILHMLDI	0.167	27	1.50E-04
